# Characteristics, treatments, and outcomes among patients with abdominal aortic injury in Japan: a nationwide cohort study

**DOI:** 10.1186/s13017-019-0262-1

**Published:** 2019-08-27

**Authors:** Yutaka Kondo, Hiroki Matsui, Hideo Yasunaga

**Affiliations:** 1 Department of Clinical Epidemiology and Health Economics, School of Public Health, The University of Tokyo, 7-3-1 Hongo, Bunkyo-ku, Tokyo, 1130033 Japan; 2 Department of Emergency and Critical Care Medicine, Juntendo University Urayasu Hospital, 2-1-1 Tomioka, Urayasu, Chiba Japan

**Keywords:** Abdominal aortic injury, Epidemiology, Trauma, Emergency departments

## Abstract

**Background:**

Abdominal aortic injury (AAI) is a life-threatening condition that occurs in only 0.1% of all trauma admissions. Because of its rarity, the clinical features of AAI remain unclear. We investigated the characteristics, treatments, and clinical outcomes among patients with AAI.

**Methods:**

This retrospective cohort study was performed using the Japanese Diagnosis Procedure Combination database. We identified patients with a confirmed diagnosis of AAI with emergency admission from 1 July 2010 to 31 March 2017. Eligible patients were divided into three groups: those who were treated with no surgery or endovascular treatment (non-repair group), those who underwent surgery without endovascular treatment (open repair group), and those who received endovascular treatment without surgery (endovascular repair group).

**Results:**

A total of 238 patients met the inclusion criteria during the study period. Of these, 191 (80.3%) were allocated to the non-repair group, 20 (8.4%) were allocated to the open repair group, and 27 (11.3%) were allocated to the endovascular repair group. The proportions of patients in the non-repair group from July 2010 to March 2012, April 2012 to March 2014, April 2014 to March 2016, and April 2016 to March 2017 were 93.5%, 75.9%, 80.6%, and 73.2%, respectively. The crude in-hospital mortality rate was 26.2%, 35.0%, and 18.5% in the non-repair, open repair, and endovascular repair group, respectively.

**Conclusions:**

In this cohort, the proportion of non-repair for AAI decreased from 2010 to 2017, whereas the proportion of endovascular repair increased. Younger patients were more likely to undergo open repair, whereas older patients were more likely to undergo endovascular repair.

## Background

Abdominal aortic injury (AAI) is a life-threatening condition. Surgery (open repair) is the standard treatment for bleeding control. AAI occurs in only 0.1% of all trauma admissions and at approximately one fifth the frequency of thoracic aortic injury [1]. Data regarding AAI are mostly based on case reports. Two cohort studies demonstrated that the overall mortality rates among patients with AAI were 35% and 51.7% [2, 3]. Because of its rarity, the clinical features of AAI remain unknown.

Non-operative treatment is becoming a treatment option for patients with AAI who have stable vital signs, even if the patients have a large intimal tear and pseudoaneurysm [4].

In recent years, endovascular repair has been rapidly adopted as a feasible treatment modality for patients with AAI because numerous studies of intrinsic disease in the abdominal aorta have shown good clinical results using endovascular stents [5, 6]. However, the role of endovascular stents in trauma settings and the features of AAI remain unclear.

The present study was performed to investigate the characteristics, treatments, and clinical outcomes among patients with AAI using a Japanese nationwide database.

## Methods

### Study design and data collection

This retrospective cohort study was performed using the Japanese Diagnosis Procedure Combination database [7]. The details of the database are described elsewhere [8]. Briefly, the database comprises administrative claims and discharge abstract data from more than 1200 acute-care hospitals in Japan [8]. It also covers approximately 90% of all tertiary-care emergency hospitals and contains the main diagnosis, primary diagnosis on admission, comorbidities present on admission, and comorbidities diagnosed during each episode of hospitalization recorded using International Classification of Diseases and Related Health Problems, 10th Revision (ICD-10) codes with text data in Japanese. A validation study for the database showed high specificity of recorded diagnoses and high sensitivity and specificity of recorded procedures [9].

### Study participants

Data recorded from 1 July 2010 to 31 March 2017 in the database were used for the present study. We studied patients with a confirmed diagnosis of AAI with emergency admission. We identified diagnoses of AAI with the ICD-10 code S350. We excluded patients who were younger than 18 years of age, died in the emergency room, and underwent both open and endovascular repairs. Eligible patients were divided into those who were treated non-operatively (non-repair group), those who underwent surgery without endovascular treatment (open repair group), and those who underwent endovascular treatment without surgery (endovascular repair group).

### Variables and outcomes

For this study, we examined the following patient background characteristics: age, sex, body mass index (kg/m^2^), Japan Coma Scale (JCS) score, coexisting injury (head injury, lumbar spine and/or pelvic fracture, bowel injury, splenic injury, and liver and/or biliary tract injury), and modified ICD-10–based Injury Severity Score (modified ICISS) [10]. A high modified ICISS indicates high severity. This score achieved high accuracy (area under the curve, 0.887) for mortality prediction among patients with trauma in the database.

Age was categorized into 18 to 49, 50 to 64, 65 to 79, and ≥ 80 years. The JCS score was categorized into four groups: 0 (alert), 1 to 3 (delirium), 10 to 30 (somnolence), and 100 to 300 (coma) points. The JCS score is well correlated with the Glasgow Coma Scale score, and a JCS score of 100 is equivalent to a Glasgow Coma Scale score of 6 to 9 [11, 12].

The primary outcome was in-hospital mortality. The secondary outcomes were 24-h mortality, length of stay, volume of blood transfusion, and major complications.

### Statistical analysis

Continuous variables are presented as medians and interquartile ranges. Categorical variables are presented as numbers and percentages. Baseline characteristics and crude outcomes were compared using the Kruskal–Wallis test for continuous variables with a skewed distribution and the chi-squared test or Fisher’s exact test for categorical variables among the non-repair, open repair, and endovascular repair groups.

We examined the numbers and proportions of non-repair, open repair, and endovascular repair from 1 July 2010 to 31 March 2012, from 1 April 2012 to 31 March 2014, from 1 April 2014 to 31 March 2016, and from 1 April 2016 to 31 March 2017.

The two-sided significance level for all tests was *P* < 0.05. All analyses were performed using Stata/SE version 15 (StataCorp, College Station, TX, USA).

## Results

**Fig. 1:**
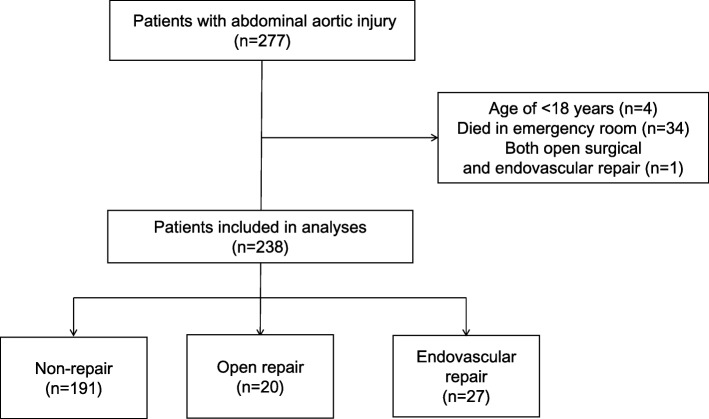
Study flow diagram of included patients

**Fig. 2:**
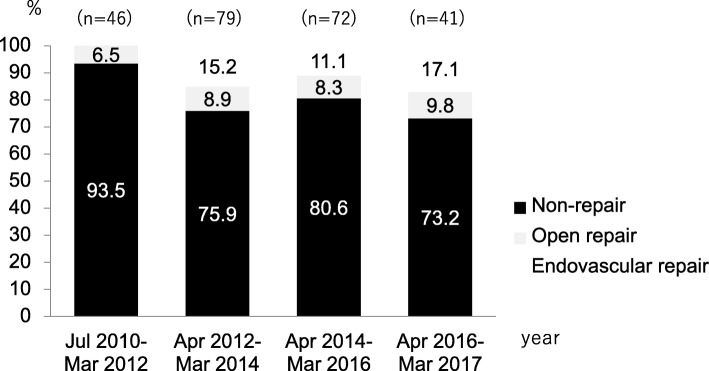
Changes and proportions of non-repair, open repair, and endovascular repair among patients with abdominal aortic injury in this study cohort

A total of 238 patients met the inclusion criteria during the study period. Of these, 191 (80.3%) underwent non-repair, 20 (8.4%) underwent open repair, and 27 (11.3%) underwent endovascular repair (Fig. 1). The numbers and proportions of patients in the non-repair group from July 2010 to March 2012, April 2012 to March 2014, April 2014 to March 2016, and April 2016 to March 2017 were 43 (93.5%), 60 (75.9%), 58 (80.6%), and 30 (73.2%), respectively (Fig. 2). The numbers and proportions of patients in the endovascular repair group during these four periods were 0 (0.0%), 12 (15.2%), 8 (11.1%), and 7 (17.1%), respectively.

The patients’ baseline characteristics are shown in Table 1. The proportions of patients with lumbar spine and/or pelvic fractures were significantly different among the non-repair, open repair, and endovascular repair groups. There were no significant differences in age, sex, body mass index, consciousness level, coexisting injury except for lumbar spine and/or pelvic fracture, or modified ICISS among the groups.

**Table 1:** Eligible patients’ baseline characteristics

	Non-repair (*n* = 191)	Open repair (*n* = 20)	Endovascular repair (*n* = 27)	*P* value
Age, years				0.91
18–49	41 (21.5)	6 (30.0)	5 (18.5)	
50–64	45 (23.6)	6 (30.0)	7 (25.9)	
65–79	62 (32.5)	5 (25.0)	8 (29.6)	
≥ 80	43 (22.5)	3 (15.0)	7 (25.9)	
Male sex	127 (66.5)	10 (50.0)	13 (48.1)	0.082
BMI, kg/m^2^	22.2 (20.1–24.9)	21.1 (19.6–24.4)	20.5 (17.7–22.5)	0.076
Consciousness level (JCS score)				0.58
0	92 (48.2)	7 (35.0)	12 (44.4)	
1–3	34 (17.8)	4 (20.0)	8 (29.6)	
10–30	27 (14.1)	3 (15.0)	4 (14.8)	
100–300	38 (19.9)	6 (30.0)	3 (11.1)	
Co-existing injury				
Head injury	18 (9.4)	0 (0.0)	3 (11.1)	0.32
Lumbar spine and/or pelvic fracture	44 (23.0)	3 (15.0)	13 (48.1)	0.016
Small intestine injury	13 (6.8)	1 (5.0)	1 (3.7)	1.0
Splenic injury	7 (3.7)	1 (5.0)	1 (3.7)	0.49
Liver and/or biliary tract injury	16 (8.4)	0 (0.0)	1 (3.7)	0.50
Modified ICISS	5.3 (4.1–7.1)	4.8 (3.5–6.2)	5.9 (4.1–7.8)	0.60

Data are presented as number (%) or median (interquartile range)

*BMI* body mass index, *JCS* Japan Coma Scale, *ICISS* International Classification of Diseases-10–based Injury Severity Score

Interventions and treatments are shown in Table 2. The proportions of patients requiring mechanical ventilation, chest tube drainage, defibrillation, and intravenous infusion (noradrenalin, dobutamine, albumin, and tranexamic acid) were significantly different among the non-repair, open repair, and endovascular repair groups.

**Table 2:** Interventions and treatments among patients undergoing non-repair, open repair, and endovascular repair

	Non-repair (*n* = 191)	Open repair (*n* = 20)	Endovascular repair (*n* = 27)	*P* value
Ventilator	64 (33.5)	15 (75.0)	17 (63.0)	< 0.001
Chest tube	18 (9.4)	3 (15.0)	8 (29.6)	0.013
Tracheostomy	11 (5.8)	3 (15.0)	4 (14.8)	0.06
Continuous renal replacement therapy	9 (4.7)	2 (10.0)	1 (3.7)	0.45
Defibrillator	4 (2.1)	3 (15.0)	2 (7.4)	0.01
Extracorporeal membrane oxygenation	2 (1.0)	1 (5.0)	0 (0)	0.26
Drugs				
Noradrenalin	31 (16.2)	10 (50.0)	8 (29.6)	< 0.001
Adrenalin	29 (15.2)	5 (20.0)	7 (25.9)	0.24
Dopamine	29 (15.2)	6 (30.0)	8 (29.6)	0.066
Dobutamine	9 (4.7)	4 (20.0)	1 (3.7)	0.036
Albumin	47 (24.6)	15 (75.0)	9 (33.3)	< 0.001
Tranexamic acid	42 (22.0)	8 (40.0)	16 (59.3)	< 0.001

Data are presented as number (%)

The outcomes are shown in Table 3. The crude mortality rate within 24 h after admission was 18.9% in the non-repair group, 15.0% in the open repair group, and 11.1% in the endovascular repair group (*P* = 0.74). The crude in-hospital mortality rate was 26.2% in the non-repair group, 35.0% in the open repair group, and 18.5% in the endovascular repair group. The median length of stay was 18.0 days in the non-repair group, 20.5 days in the open repair group, and 40.0 days in the endovascular repair group (*P* = 0.033). The blood transfusion rate was significantly different among the non-repair, open repair, and endovascular repair groups. The median volume of blood transfusion was 1440 ml in the non-repair group, 3610 ml in the open repair group, and 2240 ml in the endovascular repair group (*P* = 0.002). The proportions of pneumonia in the non-repair, open repair, and endovascular groups were 6.3%, 10.0%, and 11.1%, respectively (*P* = 0.58). The proportions of thrombosis or phlebitis in the non-repair, open repair, and endovascular groups were 2.1%, 0.0%, and 0.0%, respectively (*P* = 0.61).

**Table 3:** Outcomes among patients undergoing non-repair, open repair, and endovascular repair

	Non-repair (*n* = 191)	Open repair (*n* = 20)	Endovascular repair (*n* = 27)	*P* value
Mortality				
Died within 24 h	36 (18.9)	3 (15.0)	3 (11.1)	0.74
In-hospital mortality	50 (26.2)	7 (35.0)	5 (18.5)	0.44
Length of stay, days	18 (3–43)	20.5 (4.3–52.8)	40 (28–51)	0.033
Blood transfusion				
RBC	81 (42.4)	17 (85.0)	24 (88.9)	< 0.001
FFP	59 (30.9)	17 (85.0)	22 (81.5)	< 0.001
Platelet	30 (15.7)	12 (60.0)	12 (44.4)	< 0.001
Volume of blood transfusion, ml	1440 (560–3165)	3610 (2085–6865)	2240 (840–4068)	0.002
Complications				
Hospital-acquired pneumonia	12 (6.3)	2 (10.0)	3 (11.1)	0.58
Thrombosis/phlebitis	4 (2.1)	0 (0.0)	0 (0.0)	0.61

Data are presented as number (%) or median (interquartile range)

*RBC* red blood cells, *FFP* fresh frozen plasma

## Discussion

The present nationwide cohort study showed no significant differences in in-hospital mortality and major complications among the non-repair, open repair, and endovascular repair groups. However, the length of stay and proportion of blood transfusion showed significant differences.

These results suggest that patients in the open repair and endovascular repair groups had more severe clinical conditions because they were significantly more likely to require mechanical ventilation, chest tube drainage, defibrillation, and intravenous infusion for treatment of AAI. However, they might have also had different injury patterns necessitating other treatments. In-hospital mortality was not different among the non-repair, open repair, and endovascular repair groups.

Treatment choices are affected by the situation, environment, and surgeon. Of course, the AAI grade and injury location also affect treatment decisions.

The endovascular repair group had a prolonged length of stay. This may have been because patients who underwent endovascular repair were more likely to have pelvic fractures, which require a longer duration of bed rest. The total hospitalization costs were highest in the endovascular repair group.

Our results showed a higher proportion of lumbar spine and/or pelvic fracture in patients with AAI, which corresponds to the results of previous studies [13, 14]. These fractures are generally caused by high-energy trauma, which affects not only the lumbar spine or pelvis but also the local abdominal aorta. Thus, particular attention should be paid to the abdominal aorta on initial computed tomography imaging for patients with abdominal trauma in the emergency department.

Our results also demonstrated a higher proportion of endovascular repair in patients with AAI who had lumbar spine and/or pelvic fractures. This may have been because endovascular repair can treat both AAI and other types of bleeding.

The present study showed that the proportions of patients undergoing endovascular repair gradually increased from 2010 to 2017, whereas the proportion of patients who underwent no repair decreased (Fig. 2). These results are consistent with those in recent observational studies on thoracic aortic injury [15, 16]. In the present study, the proportion of open repair was higher in patients with AAI aged 18 to 64 years than in those aged ≥ 65 years, whereas the proportion of endovascular repair was higher in patients aged ≥ 50 years than 18 to 49 years (Table 1). These trends suggest that the proportions of older patients who undergo endovascular repair and younger patients who undergo open repair are increasing. Endovascular repair can be a good indication for older patients because it is less invasive; however, the long-term outcomes of endovascular repair for young patients remain unknown.

AAI is rarely seen in the clinical setting, and this study therefore provides useful information on the clinical features of patients with AAI. The Society of Vascular Surgery released a clinical practice guideline for thoracic aortic injuries, but a similar consensus regarding AAI has not been reached [4, 17]. Our results provide useful information that can contribute to the establishment of further consensus.

This study has several limitations. First, the type and degree of AAI, cause of trauma, cause of death, physiological parameters, and laboratory data were not available in the database. Second, no information was available regarding patients who died before reaching the hospital because the database contained only inpatient data. Third, this was a retrospective study, and the recorded diagnoses were less well validated than those in prospective registries. Finally, we evaluated only proven injuries because of the nature of the database.

## Conclusions

In this cohort, the proportion of non-repair for AAI decreased from 2010 to 2017, whereas the proportion of endovascular repair increased. Younger patients were more likely to undergo open repair, whereas older patients were more likely to undergo endovascular repair.

## Data Availability

The datasets used during the current study are available from the corresponding author on reasonable request.

## Abbreviations

AAI: Abdominal aortic injury
ICD-10: International Classification of Diseases and Related Health Problems, 10th Revision
ICISS: ICD-10–based Injury Severity Score
JCS: Japan Coma Scale
